# Distance from Unimodality for the Assessment of Opinion Polarization

**DOI:** 10.1007/s12559-022-10088-2

**Published:** 2022-12-29

**Authors:** John Pavlopoulos, Aristidis Likas

**Affiliations:** 1 Department of Computer and Systems Sciences, Stockholm University, Stockholm, Sweden; 2 Department of Informatics, University of Ioannina, Ioannina, Greece

**Keywords:** Natural language processing, Opinion polarization

## Abstract

Commonsense knowledge is often approximated by the fraction of annotators who classified an item as belonging to the positive class. Instances for which this fraction is equal to or above 50% are considered positive, including however ones that receive polarized opinions. This is a problematic encoding convention that disregards the potentially polarized nature of opinions and which is often employed to estimate subjectivity, sentiment polarity, and toxic language. We present the distance from unimodality (DFU), a novel measure that estimates the extent of polarization on a distribution of opinions and which correlates well with human judgment. We applied DFU to two use cases. The first case concerns tweets created over 9 months during the pandemic. The second case concerns textual posts crowd-annotated for toxicity. We specified the days for which the sentiment-annotated tweets were determined as polarized based on the DFU measure and we found that polarization occurred on different days for two different states in the USA. Regarding toxicity, we found that polarized opinions are more likely by annotators originating from different countries. Moreover, we show that DFU can be exploited as an objective function to train models to predict whether a post will provoke polarized opinions in the future.

## Introduction

Opinion polarization is defined as the extent of opposed opinions [1]. Often conceptualized as the opposite of agreement, it is operationalized as an index of dispersion while its multimodality characteristic is being disregarded [2]. By following a different path from prior work, we estimate opinion polarization by approaching agreement as the unimodality of the distribution and polarization as its lack. We propose a novel measure called distance from unimodality (DFU) that estimates the degree of multimodality on ordered ratings and correlates well with human judgment on the extent of polarized opinions. Empirical evidence shows two important findings. First, instances with non-unimodal annotations can be detected so that the incorrect aggregation of their annotations to a single ground truth binary label [3] can be avoided. Second, the measure can serve as a suitable objective function for supervised training of models that predict opinion polarization for texts that have not received any opinions. Furthermore, the authors expect that DFU will provide a valuable means for the study of cognitive-motivational mechanisms of political polarization in social-communicative contexts [4].

We have exploited the DFU score on two use cases, toxic language detection, and sentiment analysis. **Toxicity detection** concerns the classification of a text as toxic or not, where toxicity is an umbrella term for hateful, insulting, threatening, or otherwise abusive/offensive speech. Automated or semi-automated toxicity detection can assist with user-generated content moderation [5], prevent hate speech [6] and other types of abusive language [7], as well as assist in mitigation strategies [8]. **Sentiment analysis** is the task of automatically determining the valence or polarity (positive, negative, neutral) of a piece of text [9, 10]. The task however comprises many subtasks [11] and can be more broadly defined as the field where affective computing is applied for textual analysis [12]. Sentiment analysis has been applied to dynamical data, such as narrative progression [13] or election results [14] while, recently, deep learning was used to analyze the sentiment of people from the USA, over time, towards the policy measures against the recent COVID-19 pandemic [15].

We experimented with a sentiment analysis use case on tweets about COVID-19 that were posted in the USA [15]. We mechanically annotated the sentiment valence per tweet and we organized the data per day so that each day would form a cluster of sentiment-annotated tweets. We computed, then, the daily DFU score over 9 months during the pandemic, for two states, vis. New York and Texas. Our study shows that the distribution of opinions was polarized (having high DFU values) on different days in these two states. By manually inspecting tweets of the days with polarized opinions, we found that the reasons behind the polarization between the two states are possibly different. We experimented also with a use case on toxic text classification. By using online comments, each of which was crowd-annotated by multiple raters, we computed the DFU score per post and found that posts with more polarized opinions were more likely to be annotated by raters from different countries. Furthermore, by employing the DFU measure as a loss function to fine-tune a pre-trained language modeling Transformer [16], we found that it is possible to predict opinion polarization for unseen texts.

### Background

Opinion polarization can be defined in different ways [17]. *Spread*, for example, measures how far away are the opinions, with farther meaning more polarized. *Dispersion* defines the standard deviation of the opinions’ distribution as an indication of polarization. *Coverage* uses diversity (e.g., non-consecutive bins) to reflect less polarization. *Regionalization* uses the number of empty bins in between filled bins, so that the more the clusters the more the polarization. *Community fracturing* appears, for example, with many endogenous subgroups. *Distinctness* measures the overlap of the distributions between opinions, with more overlap indicating less polarization (a.k.a. bimodality in political polarization studies). *Group divergence* uses groups far away while *group consensus* uses the high in-group variance to indicate an unlikely polarization. Last is *size parity*, where multiple equally-heightened peaks mean more polarization, compared to one main peak (the rest being outliers). DFU combines elements from the aforementioned ways, such as size parity, community fracturing, and regionalization, but it is not characterized in any single way. Hence, we consider DFU, presented in this study, as the tenth approach.

Sixty participants were recently asked to rate the polarization of the opinions presented in each of fifteen characteristic histograms [2]. Those histograms are presented in Fig. 2. This survey approach has put in question the human perception of opinion polarization and whether a commonsense agreement can be found. Qualitative analysis showed that the perceived polarization was associated more with opinion clustering (77%), meaning that the opinions are clustered into two or more groups, compared to distant (39%) or balanced clusters (16%). The authors introduced a new polarization index, using the weighted average of the distances of the participants’ opinions. This index outperformed baselines, such as the go-to approach of standard deviation, when measuring the root mean squared error. Our proposed DFU measure outperforms standard deviation while being unsupervised. Furthermore, it is close to the above-mentioned finding that the existence of clusters matters more than the distance of the clusters.

**Fig. 1:**
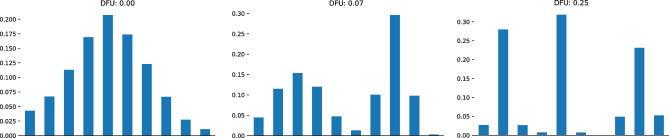
Histograms of simulated data following a unimodal normal distribution (left), a bimodal mixture of two normal distributions (middle) and a mixture of three normal distributions (right)

## Methods

### The Distance from Unimodality Measure

It is the existence of clusters in a distribution of opinions that builds the perception of polarization and not the distance of the clusters [2]. To elaborate more, consider a sentiment analysis problem with a polarity of five classes, ordered from very negative to very positive. If half raters rated the post as very positive and the rest as very negative, then this is an edge case of polarized opinions. However, if the latter had found the post neutral instead of very negative, the opinions would again remain polarized, because raters would have been divided into two clusters, but the distance between the poles would have been smaller. This observation motivates us to suggest DFU for estimating the extent of polarized opinions, more formally defined next.

Suppose a dataset $X=\{x_1, ..., x_n\}$ of *n* opinions, each of which can take *K* ordinal ratings: $x_i \in \{O^1, ..., O^K\}$. Let $f=(f_1, ..., f_K)$ be the relative frequencies of the *K* ratings defining the opinion distribution of *X*, i.e., the histogram of *X*. The discrete opinion distribution *f* is unimodal if it has a single mode. This means that there exists a maximum value $f_m$ and the values $f_i$ monotonically decrease as we move away from mode *m*. That is $f_{i-1} \le f_i$ for $i<m$ and $f_{i+1} \le f_i$ for $i>m$. Based on the definition of unimodality for discrete data, and following the same idea used in unimodality tests for continuous data [18, 19], we define DFU for an opinion histogram $f=(f_1, ..., f_K)$ as the deviation from the unimodality rule.

More specifically, let $f_m$ be the maximum value in the histogram *f*. We compute the difference values $d = (d_2, ..., d_{K})$ as follows:

1 $$\begin{aligned} d_i = \left\{ \begin{array}{ll} f_{i} - f_{i-1} &{} m< i< K \\ f_i - f_{i+1} &{} 2< i < m \\ 0 &{} i = m. \\ \end{array} \right. \end{aligned}$$

We then define *DFU* as the maximum $d_i$ value:

2 $$\begin{aligned} DFU=\max (d) \end{aligned}$$

Note that in the case of a unimodal histogram, $DFU=0$, since all $d_i$ are negative except from $d_m$ that will be equal to zero. In the case where $d_i>0$, then a deviation from the unimodality definition is found. The proposed DFU measure is defined as the maximum such deviation encountered in the histogram. For the special case of a uniform histogram, $f_{i}=\frac{1}{K}, \forall i$, hence $d_i = 0$ by definition and $DFU=0$. In other words, uniform opinion distributions are unimodal and consequently, we consider them unpolarized.

Figure 1 presents histograms with ten bins of three distributions, each simulated with 10,000 data points. The first (a) is a unimodal Gaussian distribution with a unit standard deviation. The second (b) is a mixture of two Gaussians, one with a standard deviation of three (on the left) and the other with a standard deviation of ten. The third (c) is a mixture of three Gaussians. The rightmost has a standard deviation of ten and the remaining two have a standard deviation of five). The unimodal histogram of the Gaussian (a) achieved a zero DFU score while the multimodal histograms of the latter two (b, c) achieved a positive DFU score of 0.07 and 0.25 respectively.

### Commonsense Knowledge

**Fig. 2:**
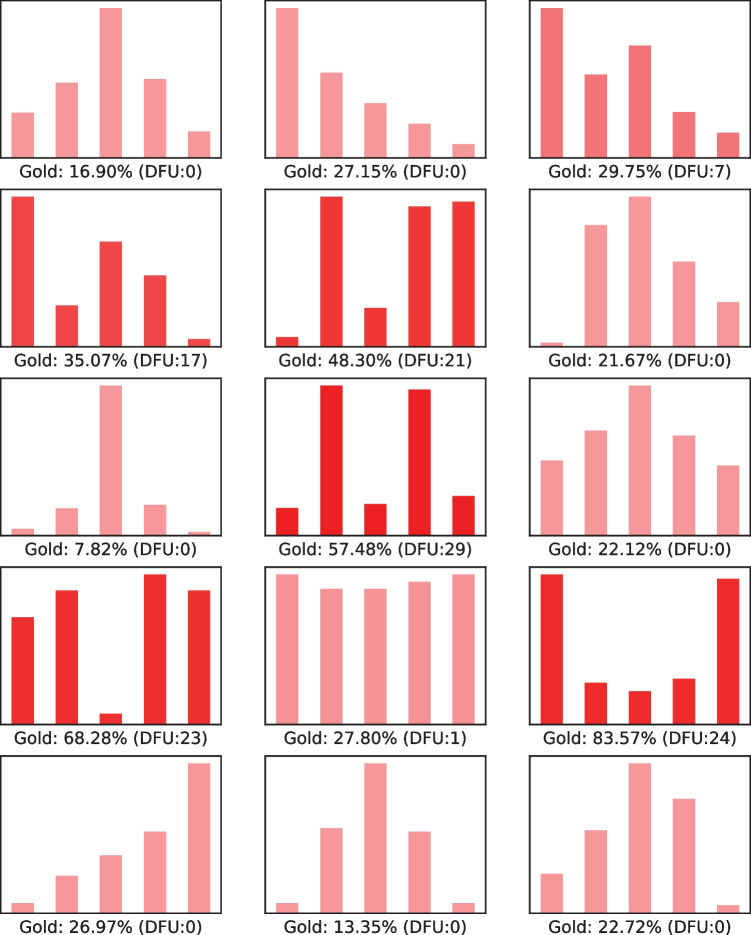
Histograms of the fifteen opinion distributions of [2]. The average judgment (percent) of the extent to which sixty polarization experts thought the respective histogram represented a polarized state is shown in the horizontal axis (Gold). Transparency is reversely related to the respective DFU score (shown in parentheses) per histogram

The opinion polarization ground truth was established in [2], where the authors approximated what humans perceive as a distribution of polarized opinions. Sixty researchers who studied opinion polarization in different fields were asked to judge the extent of polarization of a set of 15 opinion distributions on a five-point scale. We used the average judgment per distribution to build the ground truth (dubbed OPGT) regarding the extent to which the human experts thought that the respective histogram represented a polarized state. Figure 2 shows these fifteen histograms with their average judgment, along with their DFU score that will be discussed next.

**Fig. 3:**
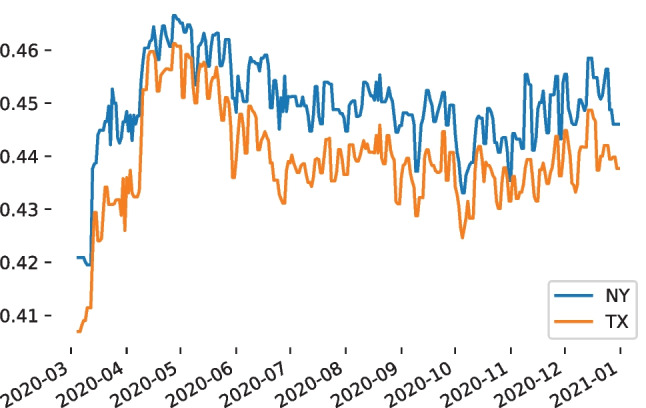
The average daily sentiment score in New York (NY) and Texas (TX) is estimated on MSTS. Scores higher than 0.61 indicate positive sentiment, ones lower than 0.43 indicate negative sentiment, and values in between indicate neutral sentiment

The sentiment of people during the pandemic was estimated over time, by using approximately five million tweets (from the USA) mentioning COVID-19-related keywords and collected from March 5, 2020, to December 31, 2020 [15]. In another study, the tweets of this dataset were re-hydrated (only IDs were shared originally) and organized per date and state [20]. The authors mechanically annotated the sentiment per tweet, by using a Transformer-based masked language model [21] that was fine-tuned to estimate the sentiment of a tweet as a valence score from zero (very negative) to one (very positive). This model achieved a root mean square error as low as 0.015 [20] on the SemEval 2018 v-reg benchmark dataset [22]. Positive is considered any tweet that was scored higher than 0.61, negative are ones that were scored lower than 0.43, and neutral ones scored otherwise [22]. We opted for this **m**echanically-annotated **s**entiment-scored **t**ime **s**eries dataset in this study (dubbed MSTS), by focusing on two states: New York and Texas. In Fig. 3, we can see that the average daily sentiment of these states is most often close to the lower limit (from 0.41 to 0.46), moving between the neutral and negative classes.

Civil Comments Toxicity Kaggle (CCTK) is a dataset comprising public comments, created from 2015 to 2017 on several English language news sites across the world.^1^ Multiple crowd-annotators per CCTK post judged the level of the post’s toxicity (i.e., “very toxic”, “toxic”, “hard to say”, “not toxic”) [23]. Polarized opinions may indicate different perceptions of the same post, for example, due to different cultural backgrounds or contexts. By contrast to MSTS, this dataset is not organized in a time series fashion.

## Results

### Correlation with Human Judgment

The correlation between DFU and human judgment may be estimated on the OPGT dataset. When comparing our DFU score against the OPGT commonsense knowledge, we report a strong correlation with human judgment: 0.91 with Spearman’s *rho* and 0.89 with Pearson’s *r*. By contrast, when we use standard deviation (dispersion), the correlation between dispersion and the ground truth across the distributions is much lower with $\rho =-0.31$ and $r=-0.12$ respectively. Noteworthy is the fact that DFU is not trained or learned on any dataset. The DFU score per histogram can be seen in Fig. 2, where higher DFU values (more opaque) are also found as of a polarized state by human experts. By experimenting with more baselines, we consistently report a correlation with the OPGT distributions that is lower than that of DFU. Leik et al. computed the dispersion as a measure of ordinal consensus for *m* categories [24], defined as: $D=\frac{2\sum _{i=1}^{m}{d_i}}{m-1}$, where $d_i$ is the cumulative relative frequency $F_i$ if $F_i<0.5$ and $1-F_i$ otherwise. The correlation was: $r=-0.62$ and $\rho =-0.58$.^2^ The $d^2$ dispersion index [25] is computed as: $d^2 = \sum _{i=1}^{k-1}{F_i-0.5}$, where $F_i$ is the cumulative relative frequency for rating category, from *i* to $k-1$. The correlation was $r=-0.36$ and $\rho =-0.38$. The kurtosis peakedness measure [26] achieves $r=-0.35$ and $\rho =-0.48$.^3^

**Fig. 4:**
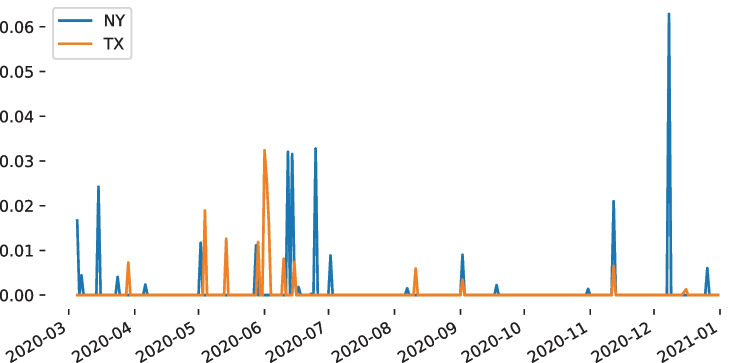
The DFU score over time for MSTS tweets from New York (NY) and Texas (TX). High peaks indicate distance from unimodality, hence at least two sentiment modes on the respective dates

### Detecting Days with Polarized Opinions

A study of days with polarized opinions was undertaken on the MSTS dataset. By focusing on New York, which had 850,897 tweets overall for 301 days, and estimating the DFU score, we found that 280 days were unimodal. Figure 4 depicts the 31 peaks, corresponding to dates when DFU was greater than zero. The highest three peaks are observed on June 6 and 25, and (the highest) on December 8 of 2020. The same study for Texas, working with 806,861 tweets on the same dates, revealed different peaks. The highest peak was on June 1st, the second highest was on June 2nd, followed by the third peak on April 5th.

### Predicting Posts with Polarized Opinions

The prediction of posts with polarized opinions was feasible on the CCTK dataset, where multiple annotators possibly from several countries judged the toxicity of each post. We used Transformers [27, 28] to fine-tune a pre-trained BERT (base) masked language model [16] for the task of predicting the outcome of our DFU measure given only a text. We trained the model on 10k posts and the same number was chosen for our development subset and our test subset. The data were organized based on time, with training posts preceding development posts, which preceded the evaluation posts. The most frequent category was “non-toxic” (68.7%), followed by “toxic” (29.4%), “very toxic” (1.5%) and “hard to say” (0.5%). DFU, which was the target variable per post, was zero for most of the posts (66%).

We used 32 posts per batch and a maximum length of 128 tokens (48 tokens on average; 75% of the data have less than 65 okens). We employed early stopping with five epochs of patience (reduce on plateau at two), we minimized the mean squared error, and we used a triangular learning policy [29] with a maximum learning rate of 1e-4, found with hyperparameter tuning.^4^ We used the implementation provided by ktrain.^5^ The fine-tuned model achieved a very low (mean absolute) error in predicting the DFU score (8.5%). Baselines performed slightly worse. A bidirectional GRU [30] on top of pre-trained word embeddings [31] achieved 9.6%. Fasttext [32] achieved 9.9%. Linear regression on top of learned word embeddings achieved 10.2%.^6^ When training the best-performing BERT to predict the dispersion, the error almost tripled (24.8%). This means that DFU can be used as a learning target, to be able to predict whether a post will provoke polarized opinions regarding its toxicity or not.^7^

## Discussion

### Sentiment Analysis

Experimental results showed that December 8th was the date when tweets in New York were most polarized (Fig. 4). The distribution during that date was bimodal and by investigating tweets from the two modes, we observed that riots and protests were a frequent cause of negative sentiment (e.g., “Stay woke people, people out here protesting, going to beaches and ect. Covid-19 is out there still...’’). On the other hand, positive tweets were related to opportunities and self-improving advice (e.g., “5 Ways Writers can Take Advantage of this Time-Opportunities in Corona Virus Chaos via...”). The date with the most polarized tweets was different for Texas, which means that the reasons behind the two polarized distributions were either different or the same but shifted in time. A manual evaluation of tweets of that date revealed that fearful sentiments due to the pandemic (e.g., “And continues to kill. Covid spike in 2 weeks.”) co-existed with optimistic content (e.g., “So yes, COVID is real. I’m blessed and thankful to have made it alive.”).

### Toxicity Classification

We also focused on CCTK posts that scored high with our DFU measure, and we observed an increased number of distinct countries the respective annotators were from. In specific, we sampled 1,000 posts whose DFU score was higher than and 1,000 posts whose DFU score was lower than the 3rd quartile. Then, we computed the average number of countries across the posts of each sample and compared the two numbers. By repeating this measurement one hundred times, the average number of countries of annotators who rated posts with a high DFU score was always higher than its counterpart ($P<0.001$). Hence, when there are polarized opinions (annotations) in a post, one explanation of the disagreement might be that the annotators come from different countries. A possible reason behind this disagreement is the different cultural backgrounds among annotators from different countries. Another possible reason is context, given that some posts may refer to topics obvious to one country (e.g., events shared by the national news) but not to others. We also note that we couldn’t establish a positive correlation between DFU and the number of countries the annotators were from. This means that a high number of countries doesn’t necessarily mean polarized opinions. Vise versa, polarized opinions are not necessarily due to annotators coming from different countries.

## Conclusion

This work introduced a novel measure for the estimation of the degree of opinion polarization, called DFU, which computes the distance from unimodality and is strongly correlated with human judgment for the same task. We experimented with a dataset of tweets, which were created by users from the USA during the pandemic and which were annotated for their sentiment valence by a fine-tuned masked language Transformer. By investigating the highest DFU scores, we found that polarized opinions in New York and Texas occurred on different dates. Furthermore, we experimented with a toxicity detection dataset, by applying DFU to the crowd annotations per post. Our findings show that annotators of posts with polarized opinions were likelier to come from different countries. Finally, by using DFU as an objective function for supervised learning, we showed that a low error can be achieved, lower than trying to predict the dispersion, which indicates that DFU can be used to learn to predict posts that will provoke polarized opinions regarding their toxicity. The proposed measure may serve as a valuable means for the study of cognitive-motivational mechanisms of political polarization [4]. Also, it may facilitate the development of better machine actionable datasets in two ways. First, noise can be removed from labeled datasets, for instance, by detecting and removing examples with non-unimodal annotations, which are often aggregated into a single binary label [3]. Second, future annotation efforts may be improved by detecting the posts that are likely to receive polarized annotations. For these posts, then, more annotators can be assigned or the context (e.g., conversational) they are provided with can be enriched.

### Supplementary Information

Notebooks with the code of the experiments presented in this study are publicly available in our repository: https://github.com/ipavlopoulos/dfu.

## Data Availability

All the datasets used in this study are publicly available or accessible from our repository: https://github.com/ipavlopoulos/dfu.

## Footnotes

1 https://www.kaggle.com/competitions/jigsaw-unintended-bias-in-toxicity-classification/data

2 We re-implemented this index. The interval (instead of the ordinal) consensus, which is based on standard deviation, gives approximately the same scores.

3 We used the implementation of scipy.stats: https://docs.scipy.org/doc/scipy/reference/generated/scipy.stats.kurtosis.html, with default values for all parameters.

4 The highest learning rate corresponding to a still falling loss was chosen.

5 https://github.com/amaiya/ktrain

6 Similar hyperparameter tuning led to a learning rate of 1e-3 for GRU, 1e-2 for fastText, and 1e-3 for linear regression.

7 Code and data are publicly available at: https://github.com/ipavlopoulos/dfu.

## Code Availability

The code used to undertake the experiments of this study is publicly available in our repository: https://github.com/ipavlopoulos/dfu.
